# Comparison of Adsorption/Desorption of Volatile Organic Compounds (VOCs) on Electrospun Nanofibers with Tenax TA for Potential Application in Sampling

**DOI:** 10.1371/journal.pone.0163388

**Published:** 2016-10-24

**Authors:** Lanling Chu, Siwei Deng, Renshan Zhao, Jianjun Deng, Xuejun Kang

**Affiliations:** 1 Key Laboratory of Environmental Medicine and Engineering (Ministry of Education), School of Public Health, Southeast University, Nanjing, China; 2 Key Laboratory of Child Development and Learning Science (Ministry of Education), Research Centre for Learning Science, Southeast University, Nanjing, China; 3 Suzhou Dongqi Biological Technology Co., LTD, Suzhou, China; Tsinghua University, CHINA

## Abstract

The objective of this study was to compare the adsorption/desorption of target compounds on homemade electrospun nanofibers, polystyrene (PS) nanofibers, acrylic resin (AR) nanofibers and PS-AR composite nanofibers with Tenax TA. Ten volatile organic compounds (VOCs) were analyzed by preconcentration onto different sorbents followed by desorption (thermal and solvent orderly) and analysis by capillary gas chromatography. In comparison to Tenax TA, the electrospun nanofibers displayed a significant advantage in desorption efficiency and adsorption selectivity. Stability studies were conducted as a comparative experiment between PS-AR nanofibers and Tenax TA using toluene as the model compound. No stability problems were observed upon storage of toluene on both PS-AR nanofibers and Tenax TA over 60 hours period when maintained in an ultra-freezer (−80°C). The nanofibers provided slightly better stability for the adsorbed analytes than Tenax TA under other storage conditions. In addition, the nanofibers also provided slightly better precision than Tenax TA. The quantitative adsorption of PS-AR nanofibers exhibited a good linearity, as evidenced by the 0.988–0.999 range of regression coefficients (R). These results suggest that for VOCs sampling the electrospun nanofibers can be a potential ideal adsorbent.

## Introduction

Sample pre-treatment is the key segment of chemical analysis. When volatile organic compounds (VOCs) often as low as ppbV to pptV are to be determined, pre-concentration becomes the most crucial step. Solid Phase Extraction (SPE), for any type of sampling, it is possible to make choices in the type of adsorbent material used, sampling method (pumped or diffusive), the method by which the trapped vapours are removed (or not) from the adsorbent for analysis [1]. Usually, common and popular adsorbents refer porous organic polymers like Tenax and Chromosorb [2]. As for VOCs collection, Tenax TA is one of the most widely utilized sorbent materials [3].

Tenax TA, a porous polymer of poly-2, 6-diphenyl-p-phenylene oxide, since the mid-1980s has been utilized for sampling VOCs. Though adsorbed loss of semi-volatiles has been related with stainless steel Tenax tubes, the hydrophobicity nature of Tenax TA avoids water-derived analytical issues experienced with multi-bed or carbon materials while still retaining a wide range of compound capture (C_6_–C_30_) [4–6]. However, Tenax tubes often lack thermal stability, and more importantly characteristic degradation peaks come about when adsorbents such as Tenax are thermally desorbed and reutilized. Usually, degraded products formed include interesting analytes, such as benzaldehyde and acetophenone from Tenax [7]. It is very little to know how Tenax TA performs in normal and extreme storage conditions, although in normal laboratory sampling conditions much effort on Tenax has been focused on analyte storage and stability. A last year’s report showed that Tenax TA thermal desorption tubes can deal with storage under environmental conditions of 4–77°C [8]. But under the common storage conditions, such as at the room temperature (20°C), refrigerator (4°C), conventional freezer (-20°C) and ultra-freezer (-80°C), Tenax TA whether or how to affect the analytical reliability is crucially probed.

SPE and Solid Phase Micro Extraction (SPME) are well developed techniques in VOCs sampling and analysis, and every method has advantages and limitations [9]. The method sensitivity of SPE can be improved by more sample volume, yet causing long sampling times. Additionally, desorption steps are often complicated and time-consuming. Therefore, it is desirable for smart pre-concentration technology of sampling VOCs to not need large sample volumes and intricate desorption procedure [10]. SPME can principally satisfy these requirements, yet its extraction efficiency relies heavily on physiochemical properties of coated fiber materials and analytes because SPME is on the basis of distribution. What’s more, its sensitivity cannot be further improved by increasing sampling volumes [11]. Considering advantages of SPE and SPME, Olesik and co-workers prepared carbon nanofibers based coatings via the pyrolysis of electrospun SU-8 nanofibers for the headspace extraction of polar and nonpolar compounds [12]. Another research group for sampling chlorobenzene developed a headspace adsorptive microextraction technique utilizing a novel polyaniline-nylon-6 nanofiber sheet manufactured by electrospinning [13]. They all confirmed that the electrospun nanofiber-based adsorbent has a potential to extract organic compounds. Recently, we demonstrated that even in the vacuum environment electrospun nanofibers exhibited good performance in sampling and gathering gaseous organic compounds [14].

As we known, electrospinning is a straightforward and versatile method for producing thin fibers, with diameters in the ranges of micrometre and nanometre, in the one-step formation of two- or three-dimensional nanofiber network assemblies composed of a broad spectrum of molecules, such as inorganic molecules as well as synthetic and biological polymers [15]. A s a recent report, the functionality of polystyrene (PS)-based nanofibers should significantly improve because of unique surface morphology. In addition, non-woven fabrics/mats of PS-based nanofibers provide the unique ability to offer a large amount of pore sizes among the adjacent nanofibers, which should enhance significantly mass transfer during the whole adsorption and desorption process [16]. This year, we continue to investigate and report the adsorption/desorption performance of VOCs onto electrospun nanofibers displayed superior than contrastive sorbent in selectivity, equilibration, regeneration and temperature effect [16]. Yet comparisons between potential sorbent (electrospun nanofibers) and most popular sampling sorbent (Tenax TA) on adsorption/desorption performance of VOCs is known very little, and further exploration of polymer nanofibers adsorbent for VOCs sampling is crucial.

In this work, we performed a comparative study of the adsorption/desorption performance of VOCs for homemade electrospun nanofibers and commercial adsorbent Tenax TA, including the characterization, adsorption amount and selectivity, desorption efficiency, precision, stability, quantitative adsorption studies. In order to gain probe into the adsorption of VOCs on polymeric nanofibers, we displayed experimental results for adsorption of typical VOCs on three structurally dissimilar polymeric adsorbents, PS nanofibers, and acrylic resin (AR) nanofibers, and PS-AR co-spinning nanofibers, which is consistent with nanofiber adsorbent in previous study [16]. And the representative VOCs, including hydrocarbon, cyclic, alcohol, ketone, ester, aromatic and chlorinated compounds, were selected as model analytes, which can suggest useful chemical information on studied sorbent phase because of different chemical properties.

## Materials and Methods

### Chemicals and Samples

All of the solvents were of analytical grade and obtained from Lingfeng Chemical Reagent Co., Ltd, Shanghai, China. Polystyrene (PS) and acrylic resin (AR) were purchased from Shanghai Chemical Agents Institute, Shanghai, China. Tenax TA (60/80 mesh) was obtained from Buchem BV (Apeldoorn, Netherlands).

### Instruments and Apparatus

The equipment referred in this research for the analysis of all target VOCs consisted of a gas chromatography-flame ionization detector (GC-FID) system (Agilent 7890A, USA). The target VOCs were separated on a HP-5 column (0.32 mm × 30 m × 0.25 μm). A high-voltage power supply (Dongwen high voltage power supply Co., Ltd., Tianjin, China) and syringe pump (Zhejiang Smith medical instrument Co., Ltd) were used for electrospinning. The nanofibers were examined using a field emission scanning electron microscope (Ultra plus SEM, Zeiss, Germany) and a specific surface and porosity analyzer (Micromeritics ASAP2020, USA). A headspace sampler (Zhongdingshichuang technology Co., Ltd, Beijing, China) was used for thermal desorption.

### Fabrication of Electrospun Nanofibers

PS, AR and PS-AR electrospun nanofibers were fabricated as following: PS of 10% (w/v) was dissolved in a mixture of dimethylformamide (DMF) and tetrahydrofuran (THF) (4:6, v/v). AR of 10% (w/v) was dissolved in a mixture of DMF and ethanol (1:4, v/v). 10% (w/v) of PS-AR (1:1, m/m) was dissolved in the mixture of DMF and THF (4:6, v/v). Next, before electrospinning these solutions were stirred for 8 h at room temperature.

**Fig 1:**
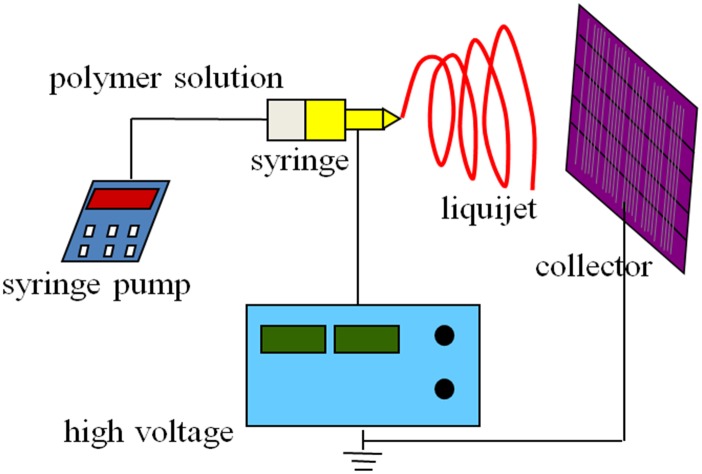
System of electrospinning.

Then every solution was loaded into the glass syringe which was fitted to a steel needle with a tip diameter of 0.5 mm. Electrospinning, shown in Fig 1, was conducted under an anodic voltage of 20 kV at 25 cm from the needle tip to collecting equipment and keeping feed rate of 2 mL/h for the precursor solution.

### Experiment Procedures

Before experiments, the tested materials were thermally conditioned to remove any residual components as follows: electrospun nanofibers were heated at 80°C for 2 hours with a nitrogen flow; Tenax TA was conditioned by thermal cleaning (100°C for 15 min, 200°C for 15 min and 325°C for 30 min) under a nitrogen flow at 100 mL/min.

### Adsorption and Desorption

**Fig 2:**
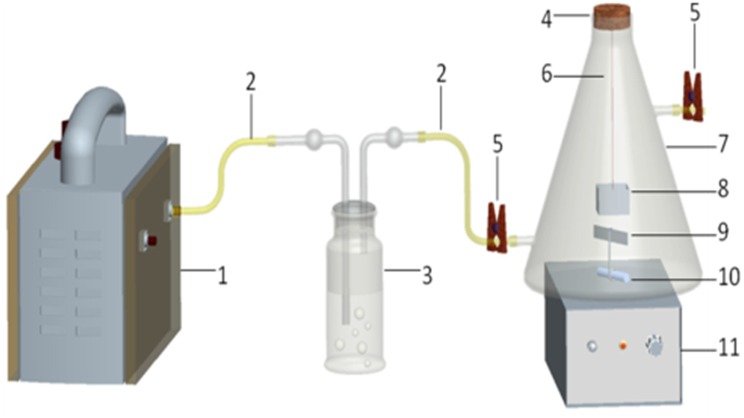
Experimental devices: 1. Vacuum pump; 2. Ventilation tube; 3. Gas cylinder; 4. Rubber plug; 5. Sealing clip; 6. Stainless steel wire; 7. Sample flask; 8. Sample box; 9. Aluminum foil; 10. Magnetic stir bar; 11. Magnetic stirrer.

As Fig 2 shown, weighed 5 mg of the adsorbent and placed it in a sample box (Fig 2, No. 8), then the sample box was hanged to the rubber plug of a 2.5 L sample flask(Fig 2, No. 7)with the stainless steel wire(Fig 2, No. 6). After all adsorbents hanging well, put all boxes into the flask at the same time, and then plug the flask immediately with the rubber plug(Fig 2, No. 4). The sample flask was connected to the gas cylinder (Fig 2, No. 3) filled with some water through which gas was generated and drawn. 1 mL of model solvent including ethanol, acetone, n-pentane, isoprene, dichloromethane, ethyl acetate, n-butanol, cyclohexane, toluene and chlorobenzene was sequentially injected into the gas cylinder to prepare a solution containing ten VOCs.

Then turned on the vacuum pump (Fig 2, No. 1), and air was drawn into the bottom of the water through the inlet ventilation tube (Fig 2, No. 2). As the air from the bottom escaped the bubbles floated to the surface and exited through the outlet ventilation tube fitting at the top of the cylinder. The target gas escaped from the liquid phase and accompanied the continuous airflow was transferred to the sample flask.

In preliminary experiments, we probed into the time of blowing gas. After 1 min, 3 min and 5 min of blowing gas, the initial peak areas of every VOC were consistent, so we chose the most convenient 1 min as the optimal time of blowing gas. Thus, after 1 min, the sealing clip was closed. The sample flask was always tightly sealed with the rubber plug. To ensure uniform distribution of the gas in the sample flask, a magnetic stirrer (Fig 2, No. 10) modified with a sheet of aluminum foil (Fig 2, No. 9) was used to speed up attainment of distribution equilibrium of VOCs during the experiment.

To verify the sample flask containing ten VOCs at the same time, the initial peak areas of ten VOCs were obtained by GC analysis of 1ml of injected sample. The tests were conducted in triplicate for every flask to indicate that every peak area variation was less than ± 5% and the process of blowing gas was prepared well.

According to the recent study on adsorption equilibration time [16], after 35 min the adsorption rate of VOCs onto the nanofibers was fast reaching adsorption equilibration during a short time. So the adsorption time set as 2 hours in this work was more than enough. Thus, after 2 hours, the tested sample materials were transferred to previously cleaned headspace vials. Considering lower temperature tolerance of nanofibers and consistent experiment conditions, all tested materials were desorbed at 80°C for 30 min. Then, 1 mL of target gas was injected into GC for analysis. After thermal desorption, the nanofibers were desorbed by 0.5 mL of methanol for 30 min. Tenax was desorbed with 0.5 mL of the mixture of acetone/hexane 1:1 (v/v) [17].

The sample components of desorption were determined using external standard quantitation. External standard calibration curves for samples were set up by preparing standards in methanol or in a mixture of acetone/hexane 1:1 (v/v) in a concentration range of 1–100 μg/mL. Then, 1 μL of the liquid was injected into GC for analysis. The determination was performed in triplicate for each sample.

### GC Conditions

The desorbed VOCs were analyzed by GC-FID. The column oven temperature of GC was programmed to increase from an initial 40°C, which was maintained for 3 min, followed by an increase to 120°C at 5°C/min and a second increase to 250°C at 20°C/min, which was maintained for 5 min. The injection temperature and detection temperature were 250°C and 280°C respectively. Nitrogen was selected as carrier gas with flow rate of 0.5 mL/min. All injections were conducted in the split mode with the split ratio of 19:1.

## Results and Discussion

### Characterization

**Fig 3:**
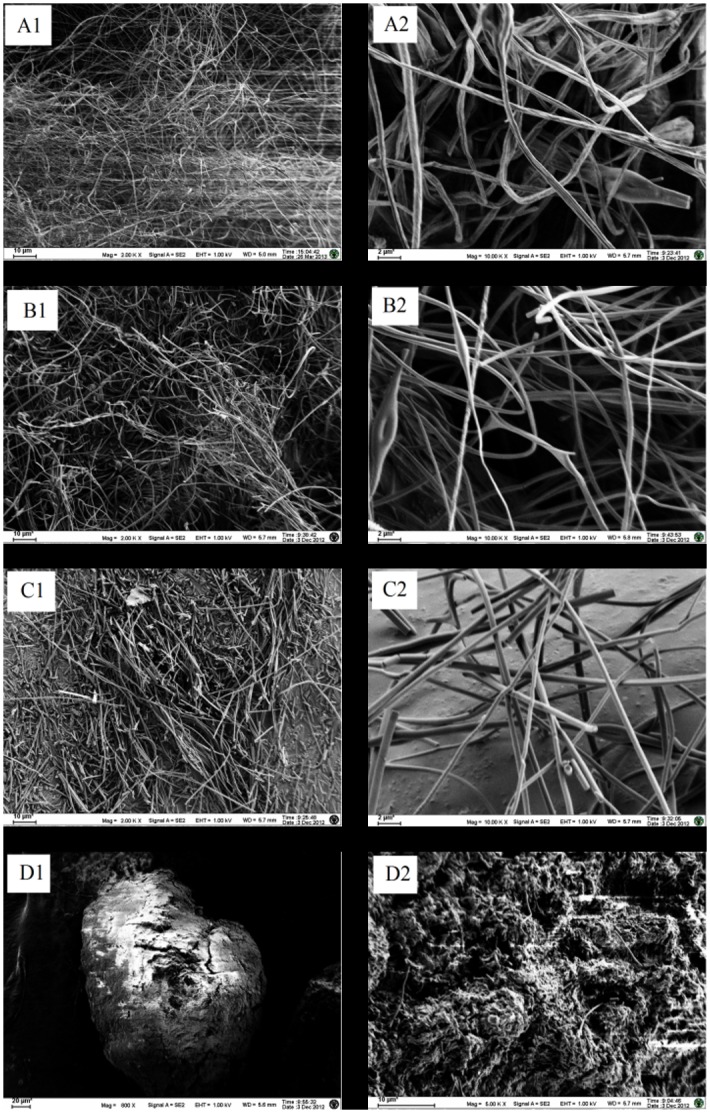
SEM images: (A) PS nanofibers (A1, 2K×; A2, 10K×); (B) AR nanofibers (B1, 2K×; B2, 10K×); (C) PS-AR nanofibers (C1, 2K×; C2, 10K×); (D) Tenax TA (D1, 800×; D2, 5K×).

The surface characteristics of tested materials were studied utilizing SEM. As Fig 3 shown, their diameters are in the range of 300–600 nm for PS nanofibers, 200–400 nm for AR nanofibers and 200–600 nm for PS-AR nanofibers. AR and PS-AR nanofibers are bead-free and have a smooth morphology. The nanofibers possess unique porous structures, which should significantly increase the surface area of the fibers. These SEM results reconfirm that the non-woven fabrics/mats composed of nanofibers afford an enormous amount of pore sizes among the adjacent electrospun nanofibers, which should cause a markedly enhanced mass transfer of analytes during adsorption and desorption process. Tenax particle was several hundred microns in size and possessed a highly porous surface that would facilitate strong adsorption.

The textural property data of PS-AR nanofibers and Tenax, including the BET surface area, pore volume and pore size, was listed in Table 1. It can be seen that the BET surface area, pore volume and pore size of PS-AR nanofibers were much lower.

**Table 1:** Data of adsorption amount normalized to that of Tenax TA.

Compound	Boiling point (°C)	Nanofibers			Tenax TA
		PS	PS-AR	AR	
**Ethanol**	79	2.46	24.7	67.9	1
**Acetone**	56	0.80	3.48	16.3	1
**n-Butanol**	118	0.52	5.17	9.82	1
**Ethylacetate**	77	0.18	0.34	0.43	1
**n-Pentane**	36	0.11	0.86	1.70	1
**Isoprene**	34	0.05	0.59	2.68	1
**Dichloromethane**	69	0.05	0.53	1.37	1
**Cyclohexane**	80.7	0.83	0.17	0.02	1
**Toluene**	111	0.58	0.20	0.05	1
**Chlorobenzene**	131.7	0.62	0.60	0.19	1

The analysis of surface area and pore size shows that the BET surface area, pore volume, pore size of representative PS-AR composite nanofibers are 19.52 m^2^/g, 0.025 cm^3^/g and 5.05 nm. However, the surface area and pore size of Tenax particle that the BET surface area, pore size and pore volume is 35 m^2^/g, 2.4 cm^3^/g and 200 nm respectively, much larger than that of PS-AR nanofibers. As these textural property data shown, the BET surface area data displayed no much difference between two materials, but their pore volume exhibited a differential of approximately one hundred times and pore size showed twenty times, which suggests the pore density of nanofibers is much higher than that of Tenax particle. Due to much smaller diameter of nanofibers, all this maybe signifies pores into nanofibers are dense and shallow. These dense and shallow pores contribute to adsorbates to arrive at adsorption site more easily, that is easier adsorption and desorption, as analogous presentation in a recent report [18].

### Adsorption Amount and Selectivity

**Fig 4:**
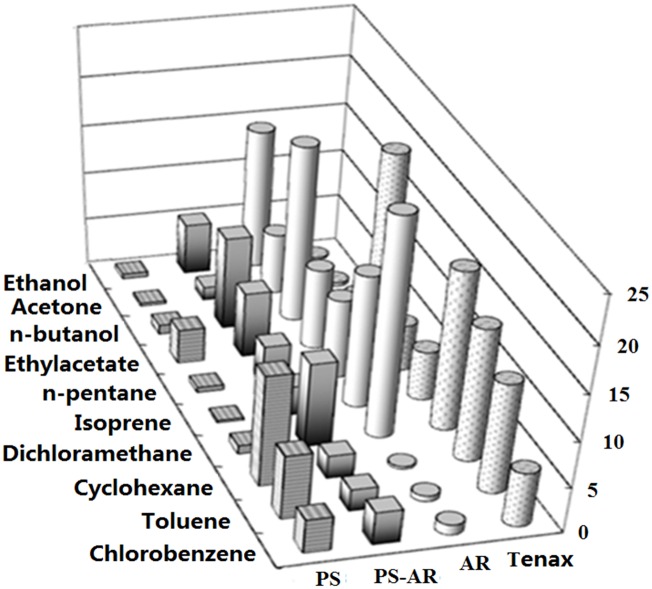
Summation (μg/5 mg) of the target VOCs by thermal and solvent desorption.

In this paper, desorption procedure was conducted in two ways, thermal desorption and solvent desorption in sequence. The adsorption amount was expressed as the sum of the mass of the targets (μg/5 mg for the sorbent) desorbed from the solid phase using two methods. The adsorption amount was shown in Fig 4, and the adsorption amount data was normalized to that of Tenax TA and listed in Table 1.

Tenax TA is one of the most widely used adsorbents for the preconcentration of VOCs even though it does not exhibit satisfactory behavior for very volatile organic compounds (VVOCs, 0 < boiling point< 50–100°C), which is primarily due to the displacement of the adsorbed volatile and polar compounds by non-polar high-molecular weight compounds [19]. As shown in Fig 4, for compounds with a higher boiling point and lower polarity, such as ethylacetate, dichloromethane, cyclohexane, toluene and chlorobenzene, Tenax TA exhibits a higher adsorption amount, and this parameter is lower for n-pentane and isoprene, is the lowest for more polar compounds, such as ethanol, acetone and n-butanol.

PS nanofibers exhibit a higher adsorption amount for toluene and chlorobenzene because the interaction between the adsorbates and adsorbent is favored by similar chemical behavior of the polymer backbone and adsorbates [20]. Therefore, specific interactions between the π-π electron rich regions of the polystyrene molecule and the aromatic rings of chlorobenzene and toluene enhanced the adsorption, that is to say, the π-π interaction provided more available adsorption sites on the surface [18]. In comparison to n-pentane, isoprene and dichloromethane, the adsorption of cyclohexane on the PS nanofibers is remarkable, but this molecule has no dipolar moment, aromatic rings or double bonds. This fact suggests that geometrical factors can play an important role in the adsorption process because cyclohexane is more circular than the other hydrocarbons studied [20–21]. It is not surprising that the presence of a polar group appears to largely reduce the adsorption of ethanol, acetone and n-butanol on the PS nanofibers. For ethylacetate, the improved adsorption on the fibers may be due to the π-π electron interaction between adsorbates and adsorbent. On the other hand, AR nanofibers performed better for the adsorption of polar molecules including ethanol, acetone, n-butanol, and ethyl acetate and worse for the adsorption of less polar molecules, such as n-pentane, isoprene, cyclohexane, toluene and chlorobenzene. However, a surprisingly high adsorption was observed for dichloromethane. The specific interaction of the dipole moment of chlorinated compounds may be involved in the adsorption process of dichloromethane on the fibers [20].

An interesting phenomenon was discovered for the PS-AR nanofibers, which exhibit an integrated adsorption behavior of PS and AR nanofibers and adsorb nearly all of the tested compounds with a broad spectrum of moderate retention. The adsorption amount of the PS-AR nanofibers for VOCs is intermediate between those of PS and AR nanofibers. This phenomenon is obviously highly dependent upon the formation of composites, which could exhibit multifunctionality, such as the π-π interactions, hydrogen bonding, polar functional groups, etc. The interaction mechanism is partly analogous to extraction of polar targets from serum, urine and other biological samples using electrospun nanofibers [22–23]. One of the advantages of electrospinning is the ability to control nanofiber composition to achieve desirable properties and functionality, as previous report that the polyacrylonitrile carbon nanofibers fabricated by electrospinning were proved as a novel alternative adsorbent [24]. Therefore, it should be possible to easily fabricate selective or broad-spectrum nanofibers sorbents by pre- and post-electrospinning modification processes. In general, a single adsorbent cannot be appropriate for the majority of compounds present in air sampling. To perform an exhaustive analysis of the air sample, adsorbents that ensure complete adsorption without the loss of sample are required [25]. It is typically good practice to combine several adsorbents to obtain improved performance. The composite nanofibers appear to be a preferable candidate for the sorbents and meet the requirements mentioned above.

### Desorption Efficiency

**Fig 5:**
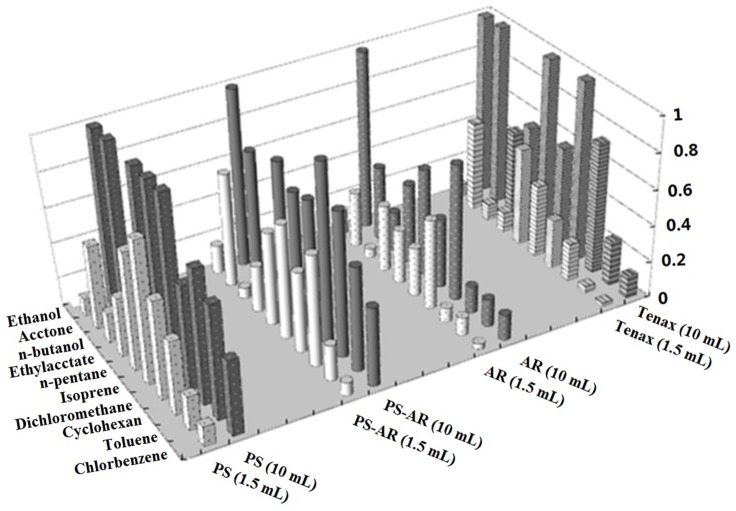
Thermal desorption rates of VOCs at 1.5 mL or 10 mL headspace volume.

An ideal sorbent for preconcentrating VOCs should have not only infinite adsorption amount for interesting compounds but also exhibit complete desorption of these compounds at moderate temperatures. During sampling the lack of retention is not the problem generally, yet polar analytes are strongly retained on most sorbents, which needs long residence times of the analytes on hot and active sorbent surface during desorption might resulting in reactions of the analytes, or reactions of the analytes with the surface itself, or reactions of the analytes with other adsorbed species. These reactions can cause permanent adsorption or/and artifact formation which is clearly undesirable effects [26]. Therefore, a good adsorbent should exhibit highly favorable adsorption as well as rapid desorption under relatively low temperature. The thermal desorption temperature was set at 80°C for this work so as to compare the differences between the tested objects, and another reason is the lower temperature tolerance of tested nanofibers. As Fig 5 shown, the thermal desorption rates of the VOCs are expressed as the proportion of VOCs released from thermal desorption over that from the sum of thermal and solvent desorption for the ten tested analytes at two different headspace volumes, 1.5 mL and 10 mL. The thermal desorption rates of the VOCs normalized to that of Tenax TA are listed in Table 2.

**Table 2:** Data of thermal desorption rates of VOCs normalized to that of Tenax TA.

Compound	1.5 ml vial				10 ml vial			
	PS	PS-AR	AR	Tenax TA	PS	PS-AR	AR	Tenax TA
**Ethanol**	1.44	2.36	2.16	1	1.01	1.03	1.00	1
**Acetone**	0.95	1.30	0.82	1	0.42	0.72	0.31	1
**n-Butanol**	2.04	0.67	0.54	1	0.38	0.65	0.46	1
**Ethylacetate**	3.09	2.41	3.48	1	1.79	1.43	0.80	1
**n-Pentane**	1.24	1.31	1.12	1	1.05	0.70	0.30	1
**Isoprene**	2.01	1.62	0.68	1	1.75	1.27	0.16	1
**Dichloromethane**	2.18	1.67	1.86	1	0.55	1.09	0.76	1
**Cyclohexane**	1.94	2.93	0.70	1	1.03	1.11	0.21	1
**Toluene**	4.49	4.84	2.39	1	2.56	2.34	0.62	1
**Chlorobenzene**	5.32	3.53	1.38	1	3.37	2.27	1.16	1

From Fig 5, the thermal desorption rates of VOCs increased when the volume of the headspace vial increased from 1.5 mL to 10 mL and the positive volume effect on the thermal desorption rates is more sensitive for PS and Tenax TA. From Table 2, in comparison to Tenax TA, the nanofibers exhibit higher thermal desorption rates for the most VOCs, especially for the smaller headspace vial. This higher desorption rate may be another advantage of nanofibers over Tenax TA because fast release of VOCs in a smaller empty vial at a lower temperature is useful for improving the sensitivity during the determination.

Comparative results show that the nanofibers have a high or comparative adsorption amount and efficient thermal desorption. The reasons may be the gaseous adsorption/desorption occurs at the surface of nanofibers, while for Tenax TA, adsorbed mass has to diffuse through micropores, mesopores and macropores, the diffusion path is long and intricate, which slows the rate of adsorption and desorption.

### Precision Studies

A precision study was conducted by the consecutive analysis of 5 samples executing adsorption/desorption. PS-AR nanofibers were selected in this experiment due to its broad-spectrum adsorption nature. 5 pieces of PS-AR nanofibers samples weighing 5 mg were placed in five separate sample boxes. Then, the boxes were placed into a vial filled with ten VOCs (conducted as mentioned in Experiment procedures of Adsorption and desorption). After adsorption for 2 hours, the nanofibers were desorbed with two methods including thermal desorption at 80°C and solvent desorption using 0.5 mL of methanol individually. Tenax TA was tested with the same procedure except that solvent desorption was performed with 0.5 mL of acetone/hexane 1:1 (v/v). Three replicate measurements of desorption samples were performed to obtain the peak area values, and only one measurement for thermal desorption. The precision was calculated as relative standard deviation (RSD) of the values.

As shown in Table 3, for thermal desorption, the RSD of PS-AR nanofibers fluctuated between 8.2% and 23.4%. Tenax TA performs well for the compounds with a higher boiling point such as n-butanol, toluene and chlorobenzene, but this parameter is larger for very volatile organic compounds, which was consistent with the lower adsorption amounts of the target VOCs onto Tenax TA (as shown in Fig 4). For solvent desorption, the RSD of PS-AR nanofibers fluctuates between 3.5% and 18.7%, which is remarkably better than that of Tenax TA for most VOCs. Comparing the RSD of thermal desorption with those corresponding to solvent desorption, it is remarkable that the former is suitable and stable than the latter, which may be due to the influence of the solvent matrix on the overlapping of some target peaks. It is shown that the PS-AR nanofiber performs better with higher precision than Tenax TA in adsorption/desorption for most VOCs.

**Table 3:** RSD (%) of PS-AR nanofibers and Tenax TA.

Compound	Thermal desorption		Solvent desorption	
	PS-AR	Tenax TA	PS-AR	Tenax TA
**Ethanol**	16.2	31.3	16.2	_*
**Acetone**	23.4	27.5	18.7	22.1
**n-Butanol**	8.6	12.1	15.5	15.2
**Ethylacetate**	12.9	12.8	3.5	_*
**n-Pentane**	10.9	25.8	9.1	22.1
**Isoprene**	19.2	27.3	12.6	18.6
**Dichloromethane**	19.9	26.3	15.9	23.2
**Cyclohexane**	17.2	18.3	14.8	22.1
**Toluene**	10.9	9.9	15.1	15.9
**Chlorobenzene**	8.2	8.3	12.5	13.4

*Not detected because of the overlapping of the solvent peak.

### Stability Study

**Fig 6:**
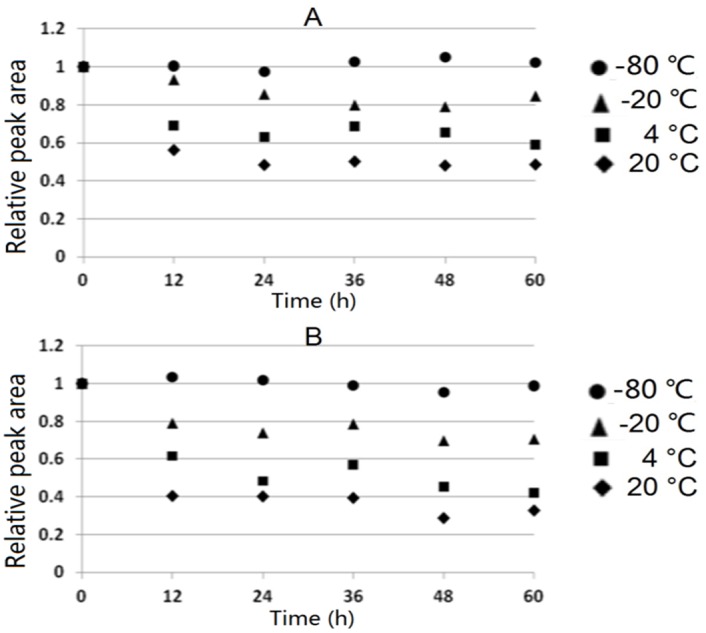
Variation in the response of samples adsorbed on (A) PS-AR nanofibers and (B) Tenax TA maintained at different temperatures.

Stability study was conducted between PS-AR nanofibers and Tenax TA using toluene as a model compound (conducted as mentioned above in adsorption and desorption). After adsorption for 2 hours, PS-AR nanofibers and Tenax TA were stored in a sealed Pyrex glass tube respectively. The tubes were respectively stored under the following conditions: room temperature at 20°C, refrigerator at 4°C, conventional freezer at -20°C and ultra-freezer at −80°C. After a specific period of time, four sets of six samples were collected at different store times to yield a total of 24 samples for each adsorbent. Then, the adsorbents were desorbed in the proper solvent as described above. Three replicate measurements of the solvent desorption liquid were performed to obtain the peak area values. The amount of toluene recovered from the adsorbents analyzed at a storage time of zero was used as the baseline for the measurement of stability. Fig 6 showed the percent recovery of toluene (expressed as a relative peak area) from the adsorbents at different storage times under various conditions.

As shown in Fig 6, the rate of desorption of toluene from both PS-AR nanofibers and Tenax TA was very fast at room temperature. However, almost no toluene desorbed when the fibers were maintained at an ultra-freezer (−80°C). It appears that the stability of the analyte adsorbed on PS-AR nanofibers is slightly better than that on Tenax TA.

### Quantitative Adsorption

The experimental device was shown in Fig 2, but the target liquid was added directly into the sample flask instead of air-blowing. Four sample flasks were needed, 0.05 mL, 0.1 mL, 0.5 mL and 1 mL of the ten targets were added separately into each sample flask containing 300 mL of water. 5 mg of PS-AR nanofibers were weighed and placed in the sample box (Fig 2, No. 8).

After 5 hours, the test materials were desorbed for 30 min in a headspace sampler at 80°C, and 1 mL of the gas sample was injected into GC for analysis.

**Fig 7:**
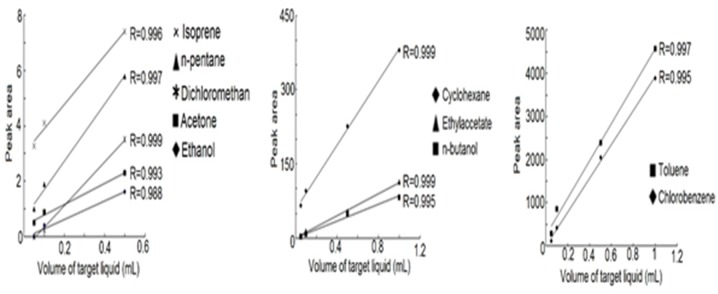
Linearity of response-amount of the target compounds in water curves.

As shown in Fig 7, with the increasing amount of the target compounds in water, the quantity desorbed from the nanofibers was increased, which also means the adsorption amount of VOCs onto the nanofibers was increased. All of the VOCs exhibited good linearity, as evidenced by the 0.988–0.999 range of regression coefficients (R) (Fig 7). Thus it can prove that the adsorption quantity of the VOCs onto the nanofibers is proportional to the concentration of the VOCs.

It is also shown that an anomalous adsorption with a narrow linear range or a larger intercept of the fitted line was observed for some VOCs. The reason is maybe that in the closed adsorption space containing ten VOCs, the interaction and interference existed between theseVOC molecules, andcompetitive adsorption and displace adsorption occurred in the adsorption sites on fibers till a dynamic adsorption equilibrium was reached.

## Conclusions

The adsorption/desorption performance of three types of nanofibers compared to that of Tenax TA, the most popular sampling material, for several VOCs was investigated. In comparison to the conventional adsorbent Tenax TA, electrospun nanofibers performed better or comparably well as the adsorbents for the VOCs tested. The electrospun nanofibers markedly displayed two special advantages. The ability to facilely fabricate selective or broad-spectrum nanofiber sorbents by pre- and post-electrospinning modification processes have been demonstrated. Additionally, the electrospun nanofibers also exhibited higher thermal desorption rates for most VOCs in the smaller empty vial at the lower temperature. These two characteristics are useful for improving the selectivity and sensitivity during VOCs sampling and determination. And the characteristics provided slightly better precision and stability of adsorbed analytes than Tenax TA. The electrospun nanofibers have been clearly suggested being a viable alternative to be the adsorbents for VOCs sampling.
